# C-N Bond Formation by Consecutive Continuous-Flow Reductions towards A Medicinally Relevant Piperazine Derivative

**DOI:** 10.3390/molecules26072040

**Published:** 2021-04-02

**Authors:** Zsolt Fülöp, Péter Bana, István Greiner, János Éles

**Affiliations:** 1 Department of Organic Chemistry and Technology, Budapest University of Technology and Economics, 1521 Budapest, Hungary; zsolt.fulop@edu.bme.hu; 2 Gedeon Richter Plc, PO Box 27, 1475 Budapest, Hungary; banap@richter.hu (P.B.); i.greiner@richter.hu (I.G.)

**Keywords:** flow chemistry, C-N bond formation, consecutive reductions, cariprazine

## Abstract

A new, continuous-flow consecutive reduction method was developed for the C-N bond formation in the synthesis of the key intermediate of the antipsychotic drug cariprazine. The two-step procedure consists of a DIBAL-H mediated selective ester reduction conducted in a novel, miniature alternating diameter reactor, followed by reductive amination using catalytic hydrogenation on 5% Pt/C. The connection of the optimized modules was accomplished using an at-line extraction to prevent precipitation of the aluminum salt byproducts.

## 1. Introduction

As a key driver of the current molecular industrial revolution [1], the modular nature of flow chemistry [2,3,4,5] facilitates the automated production of small molecules by enabling the construction of continuous-flow modules, which are responsible for the synthesis of certain structural elements [6,7,8]. Integration of such modules leads to the development of end-to-end systems [9,10,11] capable of the continuous-flow preparation of active pharmaceutical ingredients (APIs) from simple compounds, with advanced safety features and straightforward scalability [12,13,14,15,16,17,18,19]. Still, the implementation of pre-existing batch procedures can be challenging. Besides the well-known strategies to solve these limitations [20], the flow-oriented design of the synthetic route might be needed [21]. Several successful approaches have been recently described within the field of central nervous system (CNS) drugs, including the integrated continuous-flow syntheses of flibanserin [22], ketamine [23], paroxetine [24], tramadol [25], or baclofen [26].

**Scheme 1:**
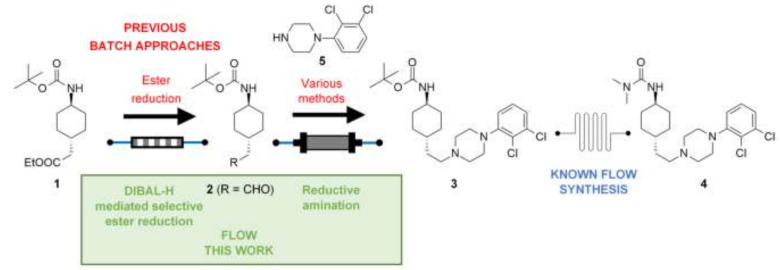
Synthetic approaches to produce the key intermediate of cariprazine, including the relevant stepwise batch methods. (DIBAL-H: Diisobutylaluminum hydride).

In the fast-evolving pharmacological toolbox of neuropsychiatry, a significant number of aminergic receptor modulating drug compounds contain the substituted 1-arylpiperazine structural unit [27]. As a recent addition to this class, cariprazine (**4**) was approved by the FDA in 2015 for the treatment of schizophrenia and bipolar I disorder [28,29]. As a continuation of our efforts to develop a continuous-flow protocol for the synthesis of this API [30], we aimed to explore the key C-N bond formation through the production of its *tert*-butoxycarbonyl protected intermediate (**3**). For this purpose, a sequence of consecutive continuous-flow reductions was designed, starting from a known ester (**1**) maintaining the beneficial trans geometry (Scheme 1).

## 2. Results and Discussion

The previously described batch procedures leading to cariprazine (**4**) obtain the key intermediate (**3**) via lengthy mesylation and *N*-alkylation steps [31,32], or utilize borane-based reducing agents [33]. For the construction of a feasible continuous-flow process (Scheme 1), we envisioned the reduction of the ester (**1**) to an aldehyde (**2**), together with a subsequent reductive amination with 2,3-dichlorophenylpiperazine (**5**) to provide the desired intermediate (**3**). 

In the continuous-flow synthesis of aldehydes from esters, the use of diisobutylaluminum hydride (DIBAL-H) has been widely reported [34,35,36,37], besides other related reducing agents, such as diisobutyl-*tert*-butoxyaluminum hydride (LDBBA) [38]. Reductive aminations also proved their utility in flow chemistry [39], especially when catalytic hydrogenation is used, which is well-suited to continuous transformations, since it is typically free from side-products, and the removal of excess hydrogen is fairly simple [40,41]. The application of reductive amination in continuous-flow hydrogenation equipment for API production has been reported in the literature [22,42]. However, to the best of our knowledge, these transformations have not been used directly after each other in a consecutive continuous-flow reaction sequence before.

To achieve high yield and selectivity during the planned ester reduction, parameters such as temperature, residence time, different reactor types, and molar ratios have been screened. In case of the reductive amination, besides optimizing for yield and productivity, selectivity was a critical factor. We identified the catalytic dehydrohalogenation of the 2,3-dichlorophenyl moiety as the main liability, which had to be addressed by the choice of the catalyst and solvent.

### 2.1. Ester Reduction Using DIBAL-H

**Figure 1:**
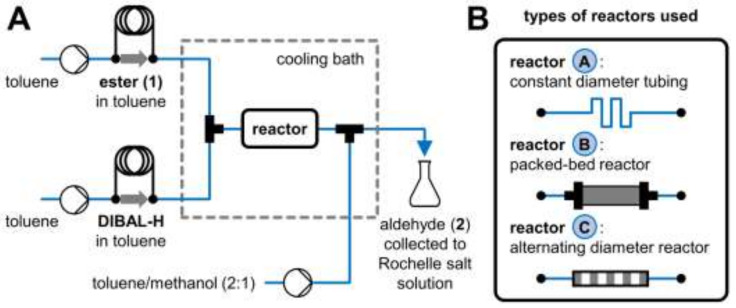
Schematic representation of the continuous-flow ester reduction module.

The reduction of the ester (**1**) to aldehyde (**2**) has been reported under batch conditions [33], which served as a foundation for our flow procedure. In the first experiments, the ester (**1**) and DIBAL-H were pumped from separate injectors, and the streams were mixed in a T-element before entering the reactor, in which simple PTFE tubing was used to provide the residence time (Figure 1, Reactor A). Then, the reaction mixture arrived at another T-shaped mixer, where it was quenched using a toluene-methanol mixture. The reactor, both T-mixers, and pre-cooling loops for each stream were submerged into a cooling bath. The product (**2**) was collected in a flask containing the saturated solution of Rochelle salt (potassium sodium tartrate), in order to solubilize the aluminum salts and simplify work-up.

Different temperatures, residence times, and reactor volumes were screened in this setup (Table 1). At 0 °C, conversion was low, and byproducts (mainly over-reduction to the alcohol) were formed in significant quantities (Entries 1–4). After decreasing the temperature to −40 °C, both conversion and selectivity improved (Entries 5–8). A slight increase in conversion was observed at longer residence times, however, it could not be further improved from a certain point (Entries 7–8), owing to the limited mixing at low flow rates. Experiments using a larger volume reactor showed no benefits in terms of conversion (Entries 9–11).

We assumed that the low conversion of the previous experiments was due to insufficient mixing. The T-shaped mixer only provides mixing at the beginning of the reactor, but it becomes ineffective in the remaining section. In order to enhance mixing, packed-bed reactors filled with inert particles can be applied [43], assuming that the reaction mixture is thoroughly mixed in the narrow channels formed between the particles. However, the flow system has to be able to handle the significant pressure drop generated on the tightly packed columns (i.e., appropriate high-pressure pumps).

The packed-beds containing inert quartz or titanium particles were introduced between the two T-mixers, in place of the PTFE tubing reactor (Figure 1, Reactor B). Different residence times were screened at −40 °C (Table 2). In case of the reactor containing quartz particles, the conversion was modest, but it could be sustained even at lower flow rates (Entries 1–3). The titanium column showed much higher conversions; however, formation of byproducts was also significant (Entries 4–6). To increase selectivity, the reaction was carried out at −70 °C, but the improvement was negligible (Entry 7). The discrepancy between the columns can be explained by slight differences in packing and particle characteristics.

**Figure 2:**
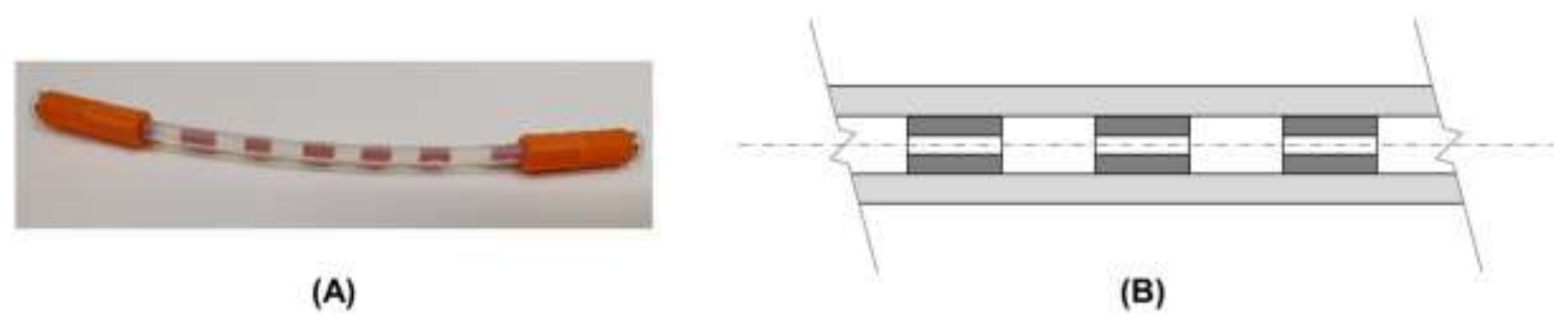
One unit of the alternating diameter reactor. Photograph of the assembled reactor (**A**) and schematic cross-section view (**B**), not to scale, end fittings are omitted). For dimensions and assembly instructions, see Supplementary Material.

Poor selectivity and the handling of the 3–5 bar pressure drop of the packed-bed reactors is not beneficial in a multistep system. Therefore, a small sized reactor with a frequently alternating inner diameter was constructed using two different types of standard microfluidic tubing (Figure 2). Owing to the frequent cross-section change, segments with turbulent flow characteristics appear, behaving like a series of mixing elements in the entire length of the reactor. This miniature device is ideal for small scale experiments with residence times inside the sub-minute range.

The alternating diameter reactor was installed between the two T-mixers (Figure 1, Reactor B), and the previous experiments were repeated at −40°C using different residence times (Table 3). An increase in conversion was observed compared to Reactor A with comparable internal volume, proving that the alternating diameter reactor provided efficient mixing. However, the previous trend that conversion increases with longer residence times needed to be reevaluated (Table 3, Entries 1–3). Apparently, the higher flow rate at shorter residence time resulted in more effective mixing, which led to higher conversion.

As we reached the optimal mixing efficiency using this type of reactor, conversion was attempted to be further increased by using a larger volume reactor (3 units of 10 cm length, connected in series) and screening different molar ratios of the reducing agent (Table 4). The excess of DIBAL-H proved to be a significant factor, leading to satisfactory conversion with high selectivity. Applying 3 equivalents of DIBAL-H at −40 °C providing 89% conversion (Entry 4) was considered as the endpoint of the optimization. Further increasing the reagent excess was detrimental to selectivity and made removal of the aluminum salts prior to the next step more inconvenient.

### 2.2. Reductive Amination

The aldehyde intermediate (**2**) was reacted with piperazine (**5**) to afford the product (**3**) under reductive amination conditions, which avoids the use of alkylating agents and bases. Catalytic hydrogenation was selected as the preferred means of reduction, which was carried out using the H-Cube^®^ reactor. The premixed solution of the purified aldehyde (**2**) and equimolar amount of piperazine (**5**) was hydrogenated at a flow rate of 0.5 mL/min on a packed-bed catalyst. With the connection of the two steps in mind, a solvent mixture of toluene-MeOH 5:1 was used, which was identical to the composition of the crude mixture exiting from the previous step after being quenched. Preliminary experiments showed acceptable selectivity using this solvent mixture (similar results were obtained using toluene), while pure methanol gave large amounts of dehalogenated byproducts.

Different temperatures and catalysts were screened, in order to keep the formation of the dehalogenated byproducts low (Table 5). Using 10% Pd/C as catalyst, the conversion was complete even at lower temperatures, however, small amounts of byproducts (mainly consisting of the dehalogenated derivatives of the product) were present (Entries 1–4). Raising the pressure (up to 50 bar) had no noticeable effect on conversion and selectivity. Excellent selectivity was observed when Raney-Ni was used as catalyst, but conversion was low even at high temperatures (Entry 5). The application of 5% Pt/C led to high selectivity at all temperatures (Entries 6–8), reaching full conversion at 80 °C (Entry 7).

As a first step towards the consecutive synthesis, the reductive amination was performed using the flow rate (2.7 mL/min) of the stream exiting the DIBAL-H reduction step. Applying the optimum conditions (5% Pt/C, 80 °C), full conversion and high selectivity were still present, despite the lower residence time. The only difference was a slight increase in pressure drop on the catalyst bed, which is normal at an elevated flow rate.

### 2.3. Two-Step Consecutive System

**Figure 3:**
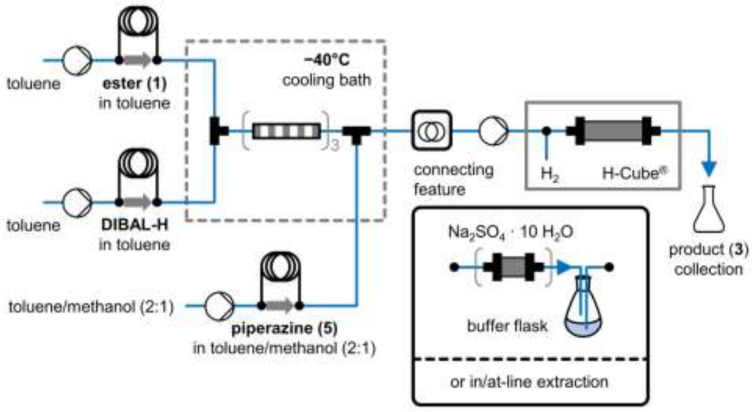
The schematic figure of the two-step consecutive system.

With the two optimized modules in hand, we aimed to connect the flow reactors and establish an integrated continuous-flow unit (Figure 3) for the preparation of the desired product (**3**). In order to simplify operation, piperazine (**5**) was introduced to the system together with the quenching agent (using an injector loop). Multiple possibilities were investigated for the realization of the connection in practice between the DIBAL-H mediated ester reduction and the reductive amination modules. This proved to be a significant challenge, given the inherent incompatibility of the two reactions.

First, the direct connection of the two steps was attempted by avoiding any form of purification or manual intervention. A buffer flask kept under nitrogen atmosphere was positioned between the two modules, the flow rate of the second reactor was adjusted to the exiting stream of the first module. However, this approach proved to be unsuccessful as clogging occurred when the reaction stream reached the 5% Pt/C catalyst column in the hydrogenation module. This can be explained by the precipitation of the dissolved aluminum salts on the surface of the catalyst, quickly blocking the flow through the catalyst bed. In order to protect the hydrogenation catalyst by the removal of the aluminum salts, a packed-bed column filled with Na_2_SO_4_ × 10 H_2_O [44] was placed on the output of the first module. We anticipated that most of the precipitation would occur on this column, but the catalyst bed still clogged after a short time.

These observations made aqueous work-up unavoidable. First, the buffer flask was filled with water to perform an in-line extraction. The reaction mixture was streamed through the water as small droplets and the organic solution was pumped from the upper phase to the hydrogenation reactor. Nevertheless, clogging developed at the same point, suggesting that a more thorough at-line extraction and careful phase separation was inevitable. For this purpose, the output of the first module was collected into a separatory funnel filled with the saturated solution of Rochelle salt. After vigorous mixing and separation, two additional extractions with Rochelle salt solution were needed, until the aqueous phase became clear, showing the complete disappearance of the aluminum salts (this kind of multi-stage extraction would not have been feasible in a continuous manner). The organic phase was directed to the hydrogenation module. The at-line extraction successfully purified the reaction stream from the aluminum salts, evidenced by the fact that clogging was not observed any longer.

Operating both modules under the previously established optimal conditions in this arrangement, the consecutive continuous-flow reduction of **1** gave the *tert*-butoxycarbonyl protected amine (**3**) with 49% yield after purification by chromatography. Scaling-out by longer operation of the same system lead to 51% isolated yield after recrystallization of the product.

## 3. Materials and Methods

### 3.1. General

Solvents and chemicals were purchased from commercial vendors. Toluene was dried over 4 Å molecular sieves for one day. DIBAL-H (1.0 M in toluene) and 2,3-dichlorophenylpiperazine (**5**) were purchased from Sigma-Aldrich (St. Louis, MO, USA). Ethyl 2-(*trans*-4-((*tert*-butoxycarbonyl)amino)cyclohexyl)acetate (**1**) was synthesized according to previously reported procedure [33]. 5% Pt/C CatCart^®^ (30 mm) was purchased from ThalesNano (Budapest, Hungary). ^1^H NMR spectra were measured on a Bruker Avance III HDX 400 MHz spectrometer equipped with ^15^N–^31^P{^1^H–^19^F} 5 mm CryoProbe Prodigy; DMSO-*d_6_* was used as solvent.

### 3.2. Procedure for the Two-Step Continuous-Flow Synthesis of Tert-butyl (trans-4-(2-(4-(2,3-dichlorophenyl)piperazin-1-yl)ethyl)cyclohexyl)carbamate (***3***)

Module 1: A system is constructed, consisting of two Asia Syringe Pumps (Syrris, Royston, UK) both having two separate flow channels, three of which are connected to two Asia Reagent Injectors (Syrris, Royston, UK, 5 mL (loop 1), 1 mL (loop 2), and 5 mL (loop 3) volume, respectively). The first two channels of the injectors are connected to a T-adaptor (Diba Omnifit^®^ PTFE T-adaptor, 1.5 mm i.d.), preceded by pre-cooling loops (0.5 mL), which is followed by an alternating diameter reactor (3 units of 10 cm long assemblies, dimensions and assembly instructions see Supplementary Material, 397.3 L net volume). The output of the reactor and the third channel are connected to a T-adaptor, followed by a second reactor (PTFE tubing, 0.8 mm i.d., 1.6 mm o.d., 0.9 mL), after which the reaction mixture is collected.

The system was washed with methanol, followed by toluene and finally anhydrous toluene. The reactors, pre-cooling loops, and T-adaptors were cooled down to −40 °C using a thermostated isopropanol bath. Washing with anhydrous toluene was upheld until steady temperature was reached. Other parts were kept at ambient temperature.

Module 2: A 5% Pt/C CatCart^®^ (30 mm) was loaded into the H-Cube^®^ (ThalesNano, Budapest, Hungary) continuous-flow hydrogenation reactor, and the system was washed with methanol, followed by a 5:1 mixture of toluene:MeOH. Reaction parameters were set to 80 °C temperature, 0.5 mL/min flow rate, and full H_2_ mode at ambient pressure. Washing with this mixture was upheld until steady state was reached.

In Module 1, anhydrous toluene was transferred on the first two channels with a flow rate of 1.25 mL/min and 187.5 L/min, respectively. Loop 1 was filled with the solution of **1** (0.05 M in anhydrous toluene), loop 2 was filled with the solution of DIBAL-H (1M in anhydrous toluene). Loop 3 was filled with the solution of **5** (0.05 M in toluene: MeOH 2:1) and the respective pump was transferring the 2:1 mixture of toluene:MeOH at 1.25 mL/min. The reaction was controlled using the Asia Manager computer program, which was responsible for switching the injector valves at appropriate times. The dead volume was discarded to the waste, until the reaction mixture appeared at the output of Module 1. Then, the reaction mixture was collected in a separatory funnel containing saturated potassium sodium tartrate solution (10 mL). The phases were shaken and carefully separated. The organic phase was extracted with further portions of saturated potassium sodium tartrate solution (2 × 10 mL). 

The organic phase was directed into Module 2, where the solution was transferred by an AZURA^®^ P4.1S (KNAUER, Berlin, Germany) HPLC pump at a flow rate (2.7 mL/min), matching that of the exiting stream from Module 1. The dead volume was discarded to the waste, until the reaction mixture appeared at the output. Then, the product mixture was collected, until the starting solution is consumed. The system was shut-down and washed with methanol.

The crude product was concentrated in-vacuo and purified by column chromatography on silica gel (EtOAc) to obtain pure **3** as white crystals (49%). When the crude product was recrystallized from acetonitrile (15 V/m), **3** was given with 51% yield. The spectroscopical data was consistent with the literature [33].

## 4. Conclusions

In conclusion, a two-step consecutive continuous-flow reduction system was established for C-N bond formation in the synthesis of a known intermediate of cariprazine (**4**). In the first ester reduction step, the main challenge was providing adequate mixing, which was achieved by the fabrication of the novel, miniature alternating diameter reactor. In case of the reductive amination, high conversion and selective transformation without dehydrohalogenation was achieved using 5% Pt/C catalyst. The consecutive two-step synthesis was accomplished with the implementation of an at-line extraction, since direct connection was unfeasible due to the precipitation of the aluminum salt content of the reaction mixture.

This method could serve as a foundation for the development of automated continuous-flow syntheses of other related drug substances, containing the 1-arylpiperazine structural unit.

## Figures and Tables

**Table 1:** Reduction using DIBAL-H in a constant diameter tube reactor (Reactor A).

Entry ^a^	Temperature [°C]	Reactor Volume [L]	Residence Time [s]	Conversion ^b^ [%]	Selectivity ^c^ [%]
1	0	20	1.2	7	32
2	0	20	2.3	6	23
3	0	20	4.6	2	10
4	0	20	9.2	3	9
5	−40	20	1.2	9	75
6	−40	20	2.3	14	68
7	−40	20	4.6	24	82
8	−40	20	9.2	23	79
9	−40	200	11.4	29	90
10	−40	200	22.9	26	86
11	−40	200	45.7	30	88

^a^ Experimental conditions: 0.05 M substrate concentration, 1 eq DIBAL-H (1M toluene solution). The flow rate of the quenching agent (toluene-MeOH 2:1) is the same as that of the substrate. ^b^ Determined by GCMS, based on TIC (Total Ion Chromatogram) product area/(product area + substrate area). ^c^ Determined by GCMS, based on TIC product area/(total area–substrate area).

**Table 2:** Reduction using DIBAL-H in packed-bed reactors (Reactor B).

Entry ^a^	Temperature [°C]	Reactor Volume ^b^ [L]	Residence Time [s]	Conversion ^c^ [%]	Selectivity ^d^ [%]
1 ^e^	−40	~250	13.8	16	56
2 ^e^	−40	~250	27.7	40	73
3 ^e^	−40	~250	55.3	44	73
4 ^f^	−40	~250	14.4	40	74
5 ^f^	−40	~250	28.8	80	64
6 ^f^	−40	~250	57.6	79	52
7 ^f^	−70	~250	14.4	50	72

^a^ Experimental conditions: 0.05 M substrate concentration, 1 eq DIBAL-H (1M toluene solution). The flow rate of the quenching agent (toluene-MeOH 2:1) is the same as that of the substrate. ^b^ Approximate dead volume (total volume–volume of the particles). ^c^ Determined by GCMS, based on TIC product area/(product area + substrate area). ^d^ Determined by GCMS, based on TIC product area/(total area–substrate area). ^e^ Packed-bed reactor (30 × 4 mm^2^ i.d.) filled with quartz particles. ^f^ Packed-bed reactor (30 × 4 mm^2^ i.d.) filled with titanium particles.

**Table 3:** Reduction using DIBAL-H in the alternating diameter reactor (Reactor C).

Entry ^a^	Temperature [°C]	Reactor Volume [L]	Residence Time [s]	Conversion ^b^ [%]	Selectivity ^c^ [%]
1	−40	200	11.4	52	86
2	−40	200	22.9	42	79
3	−40	200	45.7	38	80

^a^ Experimental conditions: 0.05 M substrate concentration, 1 eq DIBAL-H (1M toluene solution). The flow rate of the quenching agent (toluene-MeOH 2:1) is the same as that of the substrate. ^b^ Determined by GCMS, based on TIC product area/(product area + substrate area). ^c^ Determined by GCMS, based on TIC product area/(total area–substrate area).

**Table 4:** Reduction using DIBAL-H in the alternating diameter reactor with different molar ratios.

Entry ^a^	DIBAL-H eq	Temperature [°C]	Reactor Volume [L]	Residence Time [s]	Conversion ^b^ [%]	Selectivity ^c^ [%]
1	2	−40	397	21.7	56	98
2	2	−40	397	43.3	55	98
3	2	−40	397	90	39	100
4	3	−40	397	16.5	89	98
5	3	−40	397	20.7	70	94
6	4	−40	397	19.9	80	88

^a^ Experimental conditions: 0.05 M substrate concentration. The flow rate of the quenching agent (toluene-MeOH 2:1) is the same as that of the substrate. ^b^ Determined by GCMS, based on TIC product area/(product area + substrate area). ^c^ Determined by GCMS, based on TIC product area/(total area–substrate area).

**Table 5:** Reductive amination with different catalysts at various temperatures.

Entry ^a^	Temperature [°C]	Catalyst	Conversion ^b^ [%]	Selectivity ^c^ [%]	Dehalogenated Derivatives ^d^ [%]
1	30	10% Pd/C	100	97	1
2	40	10% Pd/C	98	94	3
3	50	10% Pd/C	99	93	4
4	60	10% Pd/C	100	96	2
5	100	Raney-Ni	58	93	- ^e^
6	60	5% Pt/C	69	97	- ^e^
7	80	5% Pt/C	100	97	- ^e^
8	100	5% Pt/C	97	97	- ^e^

^a^ Experimental conditions: 0.5 mL/min flow rate of the premixed substrates (0.05 M concentration), toluene-MeOH 5:1 as solvent at atm. pressure. ^b^ Determined by LCMS, based on UV-DAD product area/(product area + substrate area). ^c^ Determined by LCMS, based on UV-DAD product area/(total area–substrate area). ^d^ Determined by LCMS, based on UV-DAD (sum of the product area of mono-chlorinated and not chlorinated phenyl derivatives)/total area. ^e^ Not observed.

## Data Availability

All data presented in this study are available in the manuscript and the Supplementary Material.

## Supplementary Materials

The following are available online, Figure S1: Schematic cross-section view of the alternating diameter reactor; description of the separately operated modules; Figure S2: Photograph of the two-step consecutive continuous-flow reduction system, Figure S3: Photograph of the reactors and pre-cooling loops in Module 1, ^1^H NMR spectra of the starting material and products.

## Sample Availability

Samples of the compounds are available from the authors.
