# Supplementary material for: C-N Bond Formation by Consecutive Continuous-Flow Reductions towards A Medicinally Relevant Piperazine Derivative
Source: Molecules. 2021 Apr 2;26(7):2040. doi: 10.3390/molecules26072040 (PMC8038289; doi:10.3390/molecules26072040)
Supplement: Supplementary file 1 [file molecules-26-02040-s001.pdf]

Supplementary Material

C-N Bond Formation by Consecutive Continuous-Flow Reductions towards A Medicinally Relevant Piperazine Derivative

Zsolt Fülöp [1], Péter Bana [2], István Greiner [2] and János Éles [2,*]

[1] Department of Organic Chemistry and Technology, Budapest University of Technology and Economics, 1521 Budapest, Hungary; zsolt.fulop@edu.bme.hu

[2] Gedeon Richter Plc, PO Box 27, 1475 Budapest, Hungary; banap@richter.hu (P. B.); i.greiner@richter.hu (I. G.)

* Correspondence: J.Eles@richter.hu; Tel.: +36-1-889-8703

Table of Contents

General 2

Synthesis of the starting material 2

Construction of the alternating diameter reactor 2

Description of the separately operated modules of the two-stage continuous-flow reductions 2

Isolation of the products 3

Photographic representation of the two-step consecutive continuous-flow reduction system 4

^{1}H NMR Spectra 6

General

Solvents and chemicals were purchased from commercial vendors. Toluene was dried over 4 Å molecular sieves for one day. DIBAL-H (1.0 M in toluene) and 2,3-dichlorophenylpiperazine (**5**) were purchased from Sigma-Aldrich. 5% Pt/C CatCart® (30 mm) was purchased from ThalesNano (Budapest, Hungary). ^{1}H NMR spectra were measured on a Bruker Avance III HDX 400 MHz spectrometer equipped with ^{15}N–$^{31}P\{^{1}H$–$^{19}F\}$ 5 mm CryoProbe Prodigy; DMSO-d_6 was used as solvent.

Synthesis of the starting material

Ethyl 2-(*trans*-4-((*tert*-butoxycarbonyl)amino)cyclohexyl)acetate (1): The synthesis was performed according to previously reported procedure and the spectroscopical data was consistent with the literature [1,2].

Construction of the alternating diameter reactor

For the construction of the reactor assembly used in the optimized flow system, 3 identical pieces of PTFE tubes (1.6 mm i.d., 10 cm length each, Tube A) were employed. For a single reactor, 10 identical pieces of PEEK tubes (0.5 mm i.d., 1.6 mm o.d., 0.5 cm length each, Tube B) were placed inside Tube A at equal distances (0.5 cm) from each other. Hence, two different, alternating sections with different diameters were established in a reactor (Figure S1), ten of each, the total volume added up to 111 µl. In the optimized system, three reactors were incorporated, which were connected by two tubes (PTFE tubing, 0.8 mm i.d, 7 cm length each), thus the net volume was 397.3 µl.

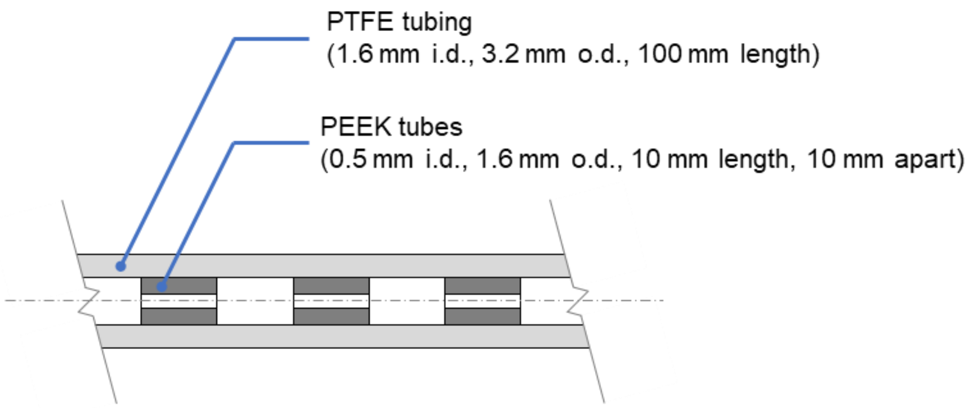

Figure S1. Schematic cross-section view of the alternating diameter reactor

Description of the separately operated modules of the two-stage continuous-flow reductions

Module 1:

Synthesis of *trans*-2-(1-(4-(*N*-*tert*-butoxycarbonyl)amino)cyclohexyl)acetaldehyde (2) by continuous-flow reduction: A system is constructed, consisting of two Asia Syringe Pumps (Syrris, Royston, United Kingdom, Pump 1) both having two separate flow channels, two of which are

connected to two Asia Reagent Injectors (Syrris, Royston, United Kingdom, 5 ml (loop 1), 1 ml (loop 2) respectively), the third channel (Pump 2) was used directly. The two channels of the injectors are connected to a T-adaptor (Diba Omnifit® PTFE T-adaptor, 1.5 mm i.d), preceded by pre-cooling loops (0.5 ml), which is followed by an alternating diameter reactor (3 units of 10 cm long, 397.3 μl net volume). The output of the reactor and pump 2 are connected to a T-adaptor, followed by a second reactor (PTFE tubing, 0.8 mm i.d., 1.6 mm o.d., 0.9 ml), after which the reaction mixture is collected. The system was washed with methanol, followed by toluene and finally anhydrous toluene. The reactors, pre-cooling loops and T-adaptors were cooled down to −40°C using a thermostated isopropanol bath. Washing with anhydrous toluene was upheld until steady temperature was reached. Other parts were kept at ambient temperature.

Anhydrous toluene was transferred on both channels of the pumps with a flow rate of 1.25 ml/min and 187.5 μl/min, respectively. Loop 1 was filled with the solution of the ester (0.05 M in anhydrous toluene), while loop 2 was filled with the solution of DIBAL-H (1M in anhydrous toluene). The flow rate of pump 2 was set at 1.25 ml/min, and the 2:1 mixture of toluene:MeOH was streamed on it. The reaction was controlled using the Asia Manager computer program, which was responsible for switching the injector valves at appropriate times. The dead volume was discarded to the waste, until the reaction mixture appeared at the output. Then, the reaction mixture was collected in a flask containing saturated potassium sodium tartrate solution (10 ml). After collection, the program was shut-down and the system was washed with toluene, followed by methanol.

Module 2:

Synthesis of the *tert*-butyl (*trans*-4-(2-(4-(2,3-dichlorophenyl)piperazin-1-yl)ethyl)cyclohexyl)carbamate (3) by continuous-flow reductive amination: A 5% Pt/C CatCart® (30 mm) was loaded into the H-Cube® (ThalesNano, Budapest, Hungary) continuous-flow hydrogenation reactor, and the system was washed with methanol, followed by a 5:1 mixture of toluene:MeOH. Reaction parameters were set to 80°C temperature, 0.5 ml/min flow rate and full H_2 mode at ambient pressure. Washing with this mixture was upheld until steady state was reached.

The solution of 23.1 mg (0.1 mmol) of **2** and 24.1 mg (0.1 mmol) of **5** in 2 ml of the mixture of 5:1 toluene:MeOH was transferred by an AZURA® P4.1S (KNAUER, Berlin, Germany) HPLC pump at 0.5 ml/min flow rate into the reactor. The dead volume was discarded to the waste, until the reaction mixture appeared at the output. Then, the product mixture was collected, until the starting solution is consumed. The system was shut-down and washed with methanol.

Isolation of the products

***Trans*-2-(1-(4-(*N*-*tert*-butoxycarbonyl)amino)cyclohexyl)acetaldehyde (2)**: The crude product was purified by column chromatography on silica gel (Cyclohexane: EtOAc 4:1) to obtain pure **2** as white crystals. The spectroscopical data was consistent with the literature [2].

Photographic representation of the two-step consecutive continuous-flow reduction system

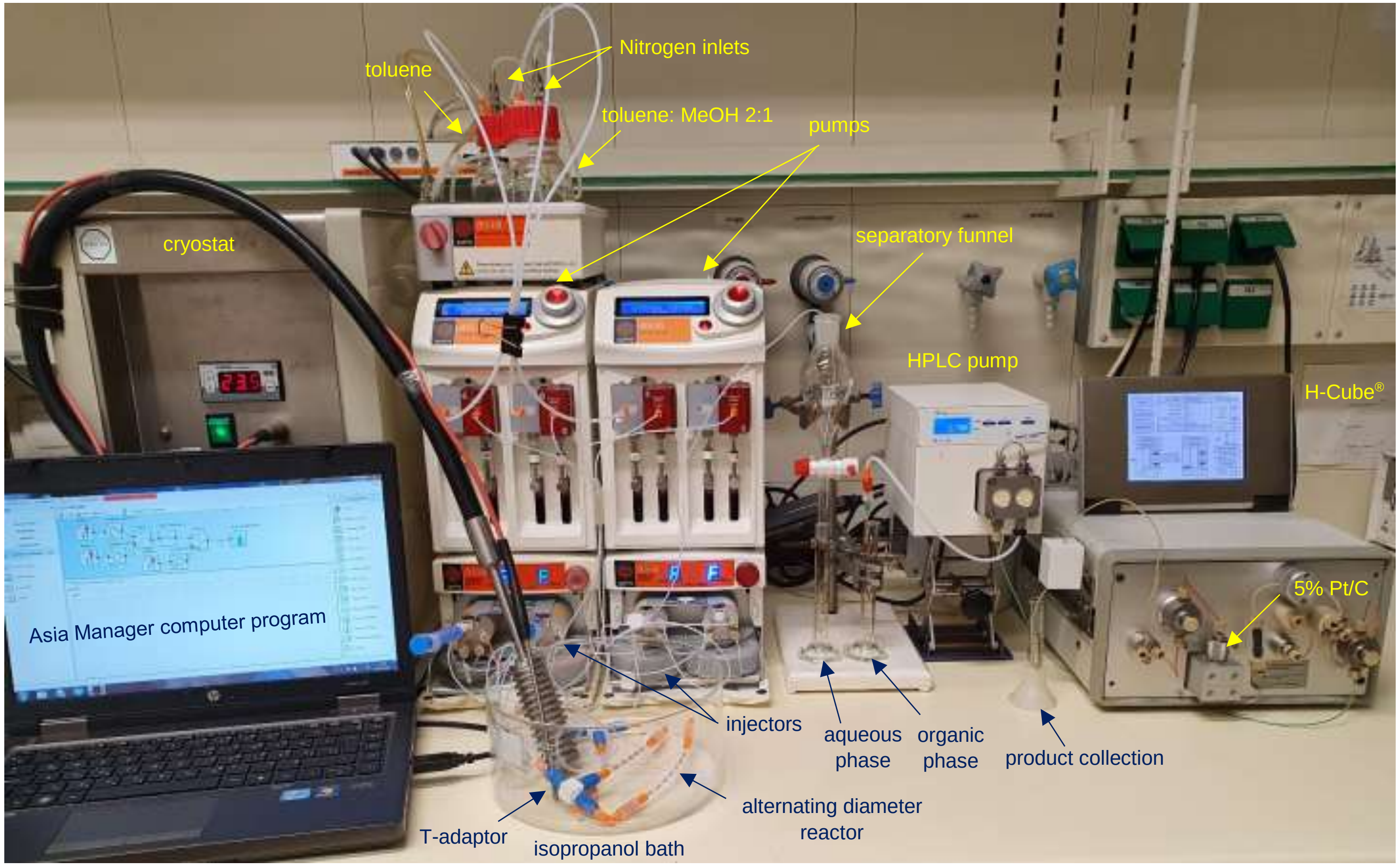

Figure S2. Photograph of the two-step consecutive continuous-flow reduction system (without cooling bath for clarity)

Photographic representation of the reactors and pre-cooling loops in Module 1

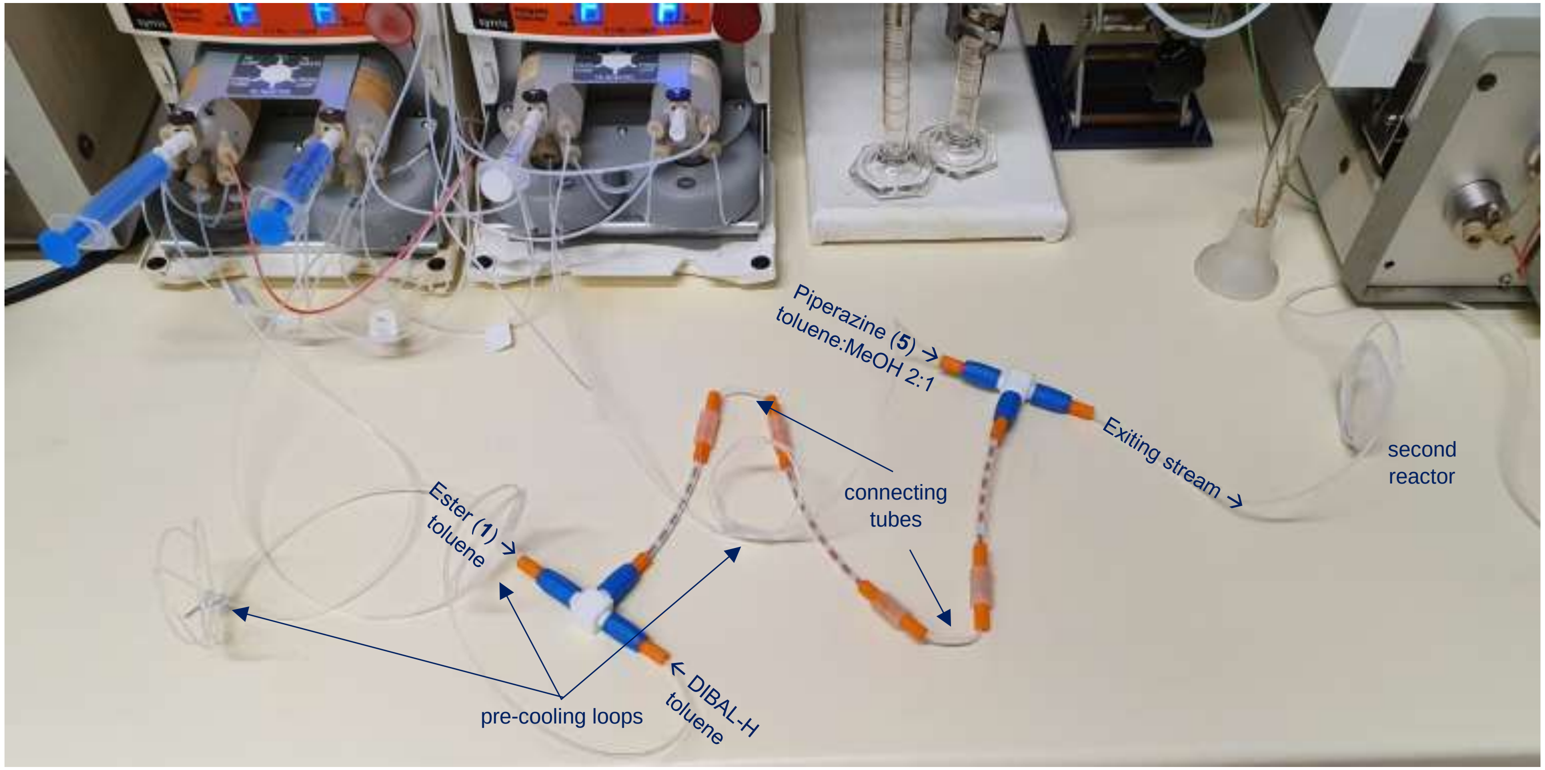

Figure S3. Photograph of the reactors and pre-cooling loops in Module 1

^{1}H NMR Spectra

Ethyl 2-(*trans*-4-((*tert*-butoxycarbonyl)amino)cyclohexyl)acetate (1)

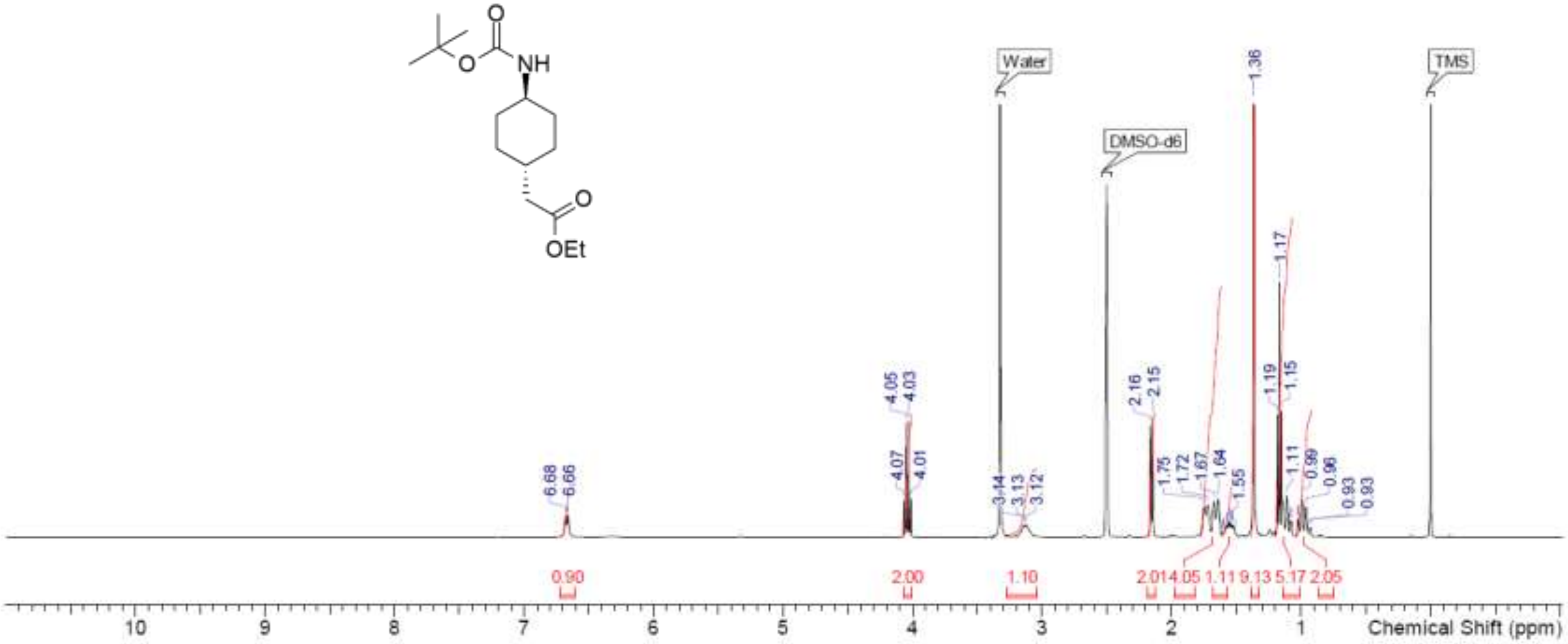

***Trans*-2-(1-(4-(*N*-*tert*-butoxycarbonyl)amino)cyclohexyl)acetaldehyde (2)**

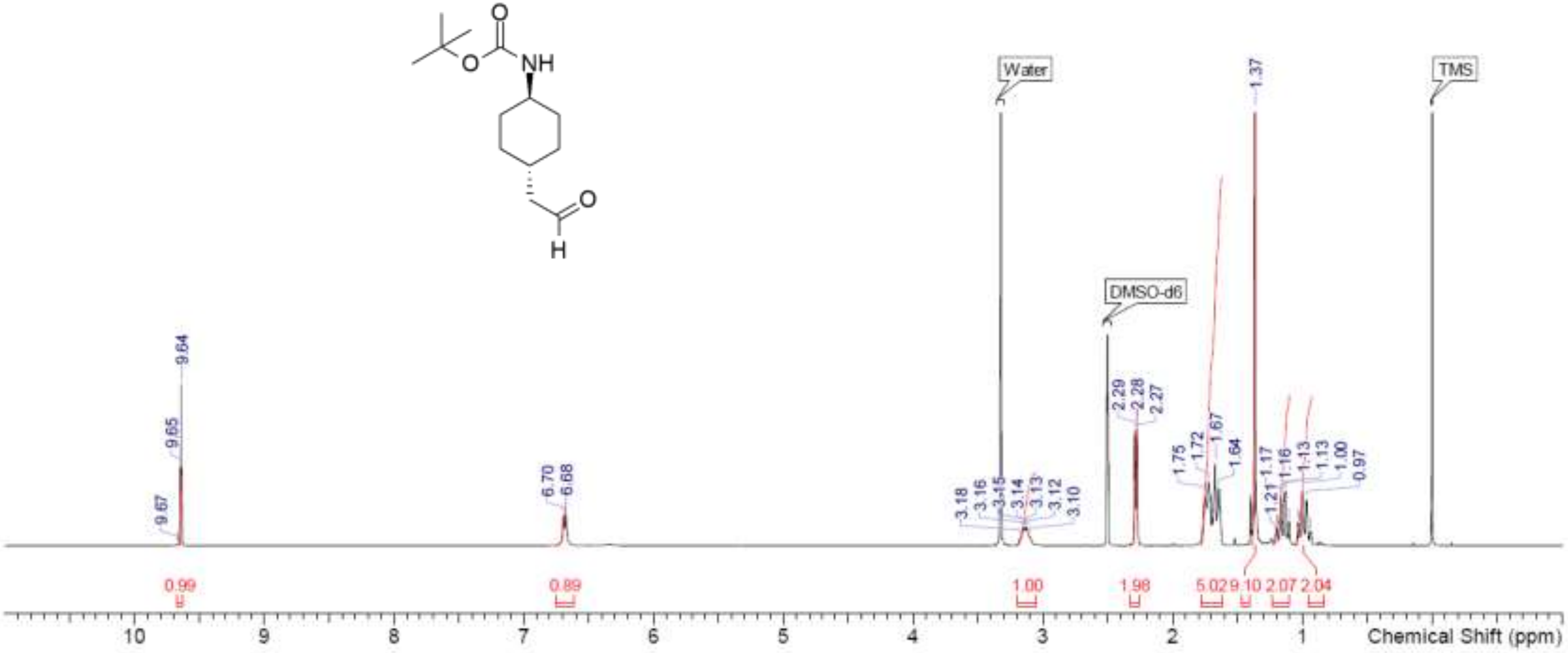

***Tert*-butyl (*trans*-4-(2-(4-(2,3-dichlorophenyl)piperazin-1-yl)ethyl)cyclohexyl)carbamate (3)**

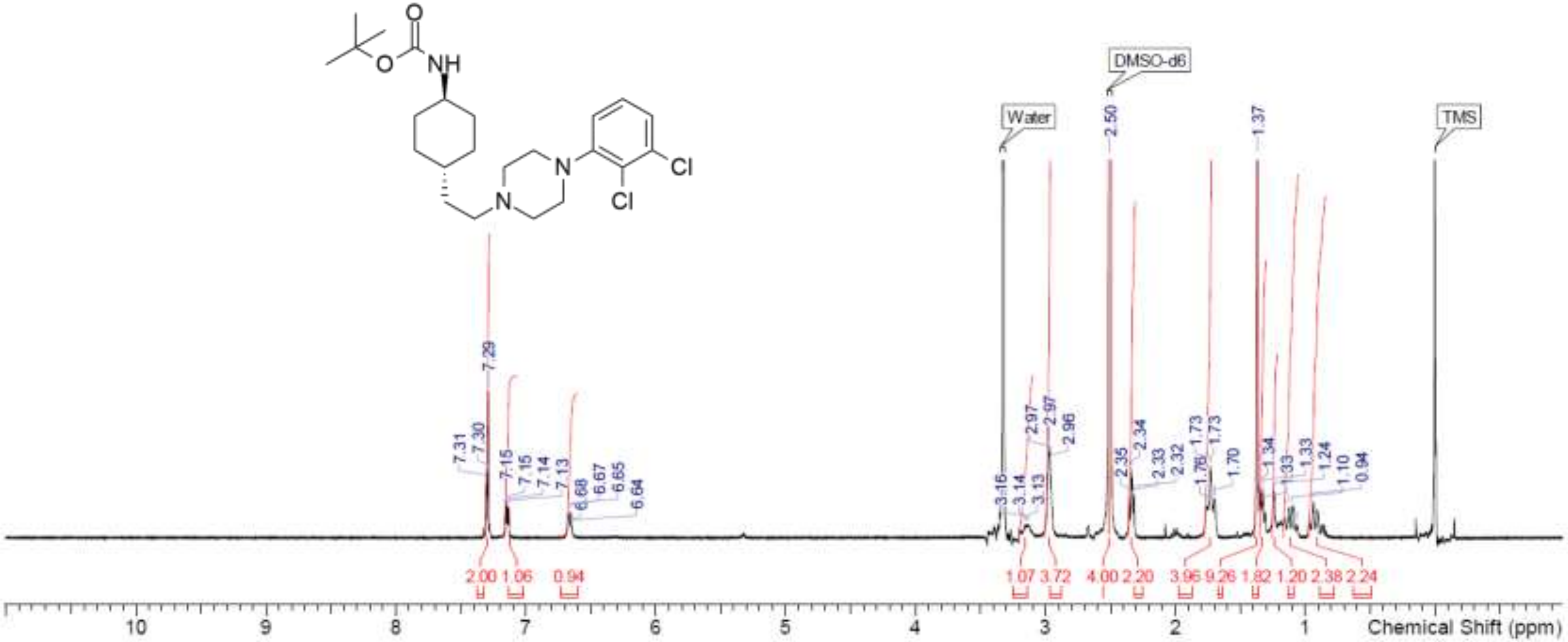

References:

1. Czibula, L.; Ágainé Csongor, É.; Nógrádi, K.; Juhász, B.; Sebők, F.; Galambos, J.; Vágó, I. Piperazine salt and a process for the preparation thereof. WO2010/070369A1, **2010**.

2. Shonberg, J.; Herenbrink, C.K.; López, L.; Christopoulos, A.; Scammells, P.J.; Capuano, B.; Lane, J.R. A Structure–Activity Analysis of Biased Agonism at the Dopamine D2 Receptor. *J. Med. Chem.* **2013**, *56*, 9199–9221.
